# Preliminary results from the EMoLung clinical study showing early lung cancer detection by the LC score

**DOI:** 10.1007/s12672-023-00799-9

**Published:** 2023-10-03

**Authors:** Karla Rubio, Jason M. Müller, Aditi Mehta, Iris Watermann, Till Olchers, Ina Koch, Sabine Wessels, Marc A. Schneider, Tania Araujo-Ramos, Indrabahadur Singh, Christian Kugler, Mircea Gabriel Stoleriu, Mark Kriegsmann, Martin Eichhorn, Thomas Muley, Olivia M. Merkel, Thomas Braun, Ole Ammerpohl, Martin Reck, Achim Tresch, Guillermo Barreto

**Affiliations:** 1 Université de Lorraine, CNRS, Laboratoire IMoPA, UMR 7365, 54000 Nancy, France; 2 Lung Cancer Epigenetic, Max-Planck-Institute for Heart and Lung Research, 61231 Bad Nauheim, Germany; 3 Universities of Giessen and Marburg Lung Center (UGMLC), Giessen, Germany; 4 German Center for Lung Research (Deutsches Zentrum für Lungenforschung, DZL), Gießen, Germany; 5 Department of Pathology, Massachusetts General Hospital and Harvard Medical School, Charlestown, MA 02129 USA; 6 International Laboratory EPIGEN, Consejo de Ciencia y Tecnología del Estado de Puebla (CONCYTEP), Instituto de Ciencias, EcoCampus, Benemérita Universidad Autónoma de Puebla, 72570 Puebla, Mexico; 7 Cologne Excellence Cluster on Cellular Stress Responses in Aging-Associated Diseases (CECAD), University of Cologne, Cologne, Germany; 8 Institute of Medical Statistics and Computational Biology, Faculty of Medicine, University of Cologne, Cologne, Germany; 9 Pharmaceutical Technology and Biopharmaceutics, Department of Pharmacy, Ludwig-Maximilians-University (LMU) Munich, 81377 Munich, Germany; 10 Comprehensive Pneumology Center Munich (CPC-M), Munich, Germany; 11 LungenClinic Grosshansdorf (GHD), Airway Research Center North (ARCN), German Center for Lung Research (DZL), 22927 Großhansdorf, Germany; 12 Asklepios Biobank für Lungenerkrankungen, Asklepios Klinik Gauting GmbH, 82131 Gauting, Germany; 13 Translational Research Unit, Thoraxklinik at Heidelberg University Hospital, 69126 Heidelberg, Germany; 14 Translational Lung Research Center Heidelberg (TLRC), 69120 Heidelberg, Germany; 15 German Cancer Research Center (DKFZ) Heidelberg, Division Chronic Inflammation and Cancer, Emmy Noether Research Group Epigenetic Machineries and Cancer, 69120 Heidelberg, Germany; 16 Institute of Pathology, University of Heidelberg, 69120 Heidelberg, Germany; 17 Department of Thoracic Surgery, University of Heidelberg, 69120 Heidelberg, Germany; 18 Department of Cardiac Development, Max-Planck-Institute for Heart and Lung Research, 61231 Bad Nauheim, Germany; 19 Institute of Human Genetics, University Medical Center Ulm, 89081 Ulm, Germany; 20 Center for Data and Simulation Science, University of Cologne, Cologne, Germany

**Keywords:** Lung cancer, Biomarker, Diagnostic, Exhaled breath condensate, GATA6, NKX2-1

## Abstract

**Background:**

Lung cancer (LC) causes more deaths worldwide than any other cancer type. Despite advances in therapeutic strategies, the fatality rate of LC cases remains high (95%) since the majority of patients are diagnosed at late stages when patient prognosis is poor. Analysis of the International Association for the Study of Lung Cancer (IASLC) database indicates that early diagnosis is significantly associated with favorable outcome. However, since symptoms of LC at early stages are unspecific and resemble those of benign pathologies, current diagnostic approaches are mostly initiated at advanced LC stages.

**Methods:**

We developed a LC diagnosis test based on the analysis of distinct RNA isoforms expressed from the *GATA6* and *NKX2-1* gene loci, which are detected in exhaled breath condensates (EBCs). Levels of these transcript isoforms in EBCs were combined to calculate a diagnostic score (the LC score). In the present study, we aimed to confirm the applicability of the LC score for the diagnosis of early stage LC under clinical settings. Thus, we evaluated EBCs from patients with early stage, resectable non-small cell lung cancer (NSCLC), who were prospectively enrolled in the EMoLung study at three sites in Germany.

**Results:**

LC score-based classification of EBCs confirmed its performance under clinical conditions, achieving a sensitivity of 95.7%, 91.3% and 84.6% for LC detection at stages I, II and III, respectively.

**Conclusions:**

The LC score is an accurate and non-invasive option for early LC diagnosis and a valuable complement to LC screening procedures based on computed tomography.

**Supplementary Information:**

The online version contains supplementary material available at 10.1007/s12672-023-00799-9.

## Introduction

Current LC diagnostic strategies include chest X-ray (CXR), low-dose helical computed tomography (CT), positron emission tomography CT (PET CT) and morphological invasive sampling. However, diagnostic approaches are often initiated at advanced stages since the majority of patients is asymptomatic at early stages of the disease. Studies implementing CT demonstrated that early diagnosis is crucial to reduce the extremely high case fatality rate of LC (95%) [1–4]. Unfortunately, CT-based LC screening approaches in high risk populations is a procedure with very high percentage of false-positive observations (> 90%) and hence low specificity (73.4%) [5], resulting in unnecessary follow-up CT scans, bronchoscopy, or even surgery [6–8]. Accordingly, there is an increasing need of employing less invasive diagnostic methods and biomarkers to complement the success of CT for LC diagnosis.

Collection of exhaled breath through cooling devices provides options for the development of non-invasive LC diagnostic methods [9–15]. Following this idea, we previously established reproducible standard operating procedures (SOPs) for a complete LC diagnosis method, consisting of EBC collection, storage, and processing for isoform-specific expression analysis [16]. We showed that RNA purified from EBCs can be used for qRT–PCR-based isoform-specific expression analysis of *GATA6* and *NKX2-1*, two genes important for embryonic lung development [17, 18] and with implications in LC [19–25]. The levels of adult and embryonic transcript isoforms from *GATA6* and *NKX2-1* were measured in EBCs and combined into one diagnostic score (LC score). The high performance of the LC score-based diagnosis was confirmed in an independent validation cohort [16]. However, the results of our previous study did not prove its usefulness under clinical conditions, for which the clinical study EMoLung was designed. Furthermore, we increased the number of early stage LC samples (I-II) in EMoLung, which was relatively low in our previous study, to determine the performance of the LC score for early LC diagnosis.

## Methods

### Study design and study population

The study was performed according to the principles set out in the WMA Declaration of Helsinki and to the protocols approved by the institutional review board and ethics committee of the University of Lübeck (AZ: 17-065). A flowchart depicting different steps of the clinical study EMoLung is represented in Fig. S1a (Supplementary Material). Patients were prospectively enrolled into EMoLung as they were undergoing diagnostic evaluation for LC, prior to surgery, at the LungenClinic Grosshansdorf GmbH (LCG), the Asklepios Klinik Gauting GmbH (ASK), and the Thoraxklinik at Heidelberg University Hospital (TKUH). After surgical intervention, cases were reviewed by an expert panel of pathologists, radiologists, pulmonologists and oncologists in the different cohorts according to the current diagnostic criteria for morphological features and immunophenotypes recommended by the International Union Against Cancer [26]. Additional inclusion criteria were (i) a non-small cell lung cancer (NSCLC) diagnosis, (ii) clinical stage I-III according to TNM classification 8th edition, (iii) patient following the recommendation of a curative tumor resection, (iv) index of the Eastern Cooperative Oncology Group (ECOG) being ≤ 2, (v) patient age ≥ 18 years, and (vi) patient having signed an informed written consent. Patients diagnosed with small cell lung cancer (SCLC) and patients receiving neoadjuvant chemotherapy or chemoradiotherapy were excluded. Patients enrolled into the EMoLung will be followed up for up to 2 years after surgical resection, in which EBCs will be collected before surgical resection, 3, 12, 18 and 24 months after surgical resection and/or at the time of recurrence. For the current study, only the base line EBCs were included. The study population is described in Fig. S1b (Supplementary Material), Table 1 and Table S1 (Supplementary Material). Briefly, the LC group consisted of 121 EBCs from 103 LC patients (99 NSCLC and 4 carcinoid), including 5 EBCs from 3 stage IV NSCLC patients to confirm previous results [16]. The control group comprised 46 EBCs from 23 donors, who either had no diagnosis of LC (36 EBCs from 13 donors), or were originally suspected to be LC patients but subsequently, pathologically diagnosed as non-malignant (10 EBCs from 10 donors).

**Table 1:** Clinical characteristics of patients

Clinical characteristic	Total Ctrl	Total LC
N	23	103
Age		
≤ 60	14 (60.87%)	25 (24.27%)
60–69	5 (21.74%)	34 (33.01%)
≥ 70	4 (17.39%)	44 (42.72%)
Gender		
Male	10 (43.48%)	62 (60.19%)
Female	13 (56.52%)	41 (39.81%)
Smoking history		
Current (CS)	13 (56.52%)	78 (75.73%)
Former (PS)	1 (4.35%)	16 (15.53%)
Never (NS)	9 (39.13%)	9 (8.74%)
LC stage		
I	–	46 (44.66%)
II	–	23 (22.33%)
III	–	27 (26.21%)
IV	–	3 (2.91%)
NA	–	4 (3.88%)
N per Center		
TKUH	2 (8.70%)	15 (14.56%)
ASK	4 (17.39%)	26 (25.24%)
LCG	14 (60.87%)	62 (60.19%)
MPI	3 (13.04%)	–
NSCLC subtypes		
AC	–	67 (65.05%)
SCC	–	28 (27.18%)
LCC	–	2 (1.94%)
ACC	–	1 (0.97%)
Undetermined	–	1 (0.97%)
No NSCLC	–	4 (3.88%)

Characteristics of the population participating in the clinical study EMoLung in the baseline phase. N refers to the number of participants in the set. Total N value in control group (Ctrl) is 23. Total N value in lung cancer group (LC) is 103. Pathological tumor stage is given according to the TNM classification 8th edition. Participating clinical centers: LungenClinic Grosshansdorf GmbH (LCG), the Asklepios Klinik Gauting GmbH (ASK) and the Thoraxklinik University of Heidelberg (TKUH). Histological subtypes of non-small cell lung cancer (NSCLC): adenocarcinoma (AC), squamous cell carcinoma (SCC)

*NA* No information

### EBC collection, gene expression analysis and LC score

EBC collection, gene expression analysis by qRT–PCR and LC score calculation were performed as previously described [16]. Briefly, EBC collection was performed using the RTube (Respiratory Research) as described online (http://www.res piratoryresearch.com/products-rtube-how.htm) and following the guidelines for EBC sampling by the ERS/ATS Task Force [27, 28]. Total RNA isolation from EBC was performed using 500 µl of sample and the RNeasy Micro Kit (Qiagen). Complementary DNA (cDNA) was synthetized using the High Capacity cDNA Reverse Transcription kit (Applied Biosystem) with 0.5–0.7 µg total RNA. RT reaction without adding enzyme was used as negative control. qRT–PCRs were performed using SYBR® Green on the Step One plus Real-time PCR system (Applied Biosystems) using the primers previously described [16]. Briefly, 1 × concentration of the SYBR Green master mix, 250 nM each forward and reverse primer, and 3.5 µl (EBC) from a sixfold diluted RT reaction were used for the gene-specific qPCR. Isoform expression values were determined by calculating 2^(-Ct-value) for each of the three technical replicate measurements and, subsequently, taking the mean of these values. Then, the Em/Ad isoform ratios of *GATA6* and *NKX2-1* were used to calculate the LC score as previously described [16]:

$$LC score=(0.715*log2\left(\frac{GATA6Em}{GATA6Ad}\right)+log2\left(\frac{NKX2.1Em}{NKX2.1Ad}\right)*0.855+1.312)$$

A sample with LC score > 0 will be classified as a lung cancer sample; otherwise, the samples are classified as control samples (see Table S8 in Supplementary Material).

### Statistical analysis

**Fig. 1:**
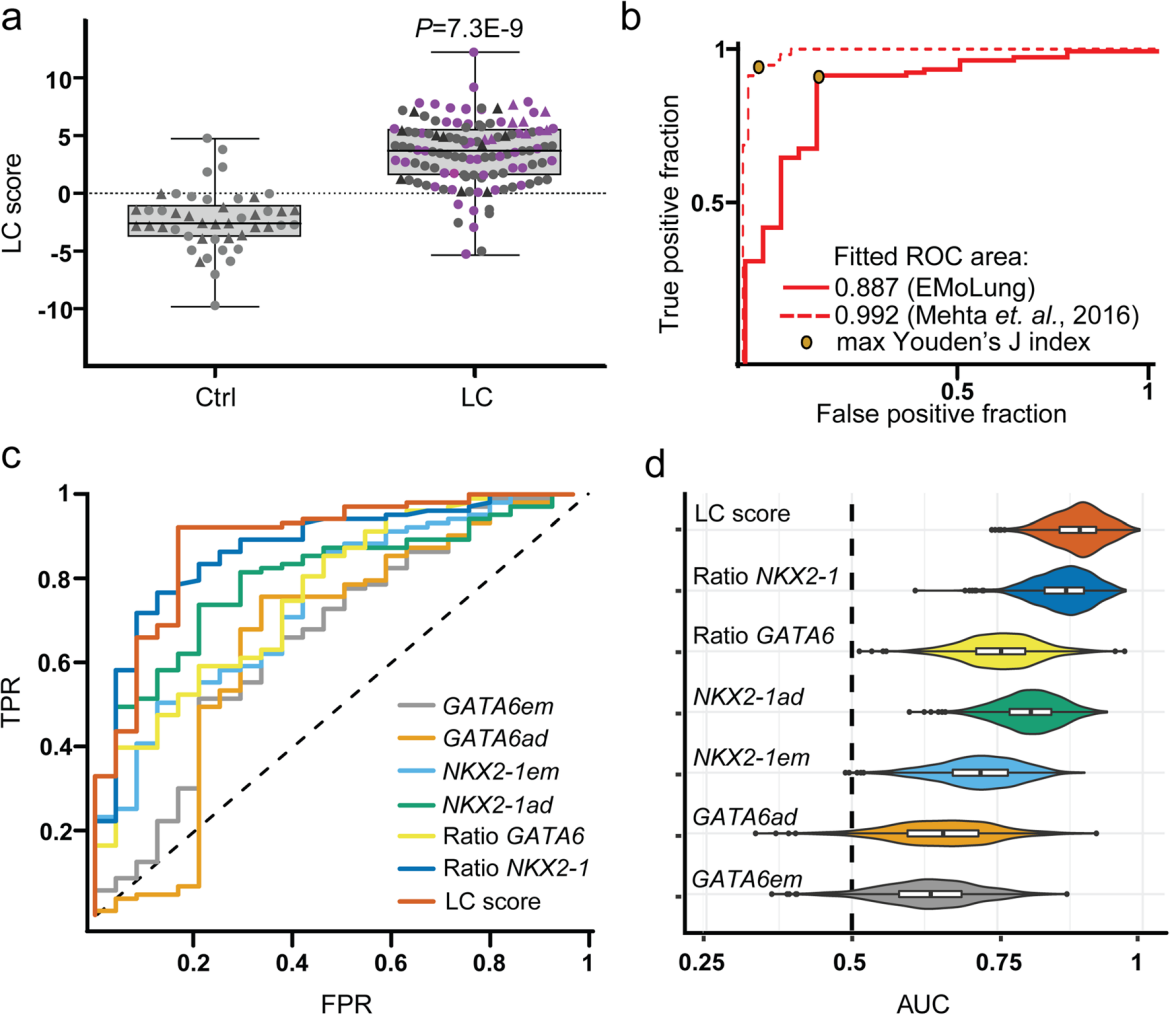
High performance of LC score-based classification of EBCs under clinical settings. **a** Box plot of the LC score detected in EBCs from control (Ctrl) and lung cancer (LC) patients. Circles represent single samples, triangles represent technical replicates. Pink circles represent LC stage I samples. *P* values relative to Ctrl were calculated by two-sided Mann–Whitney U test. The five-number summary and the statistical test values are shown in Table S1 (see Supplementary Material). **b** ROC analysis confirmed the high performance of the LC score based classification of EBCs under clinical settings (red line) compared to the classification on the validation set of EBCs (red dotted line) performed by Mehta et. al., 2016. The red line represents the ROC curve for lab 1 measurements (picking exactly one random replicate per patient if necessary). The area under the curve (AUC) values for each study are shown. The orange diamonds represent the optimal operating point of the SVM classifier, which is the point on the curve with maximal Youden’s J index. See Table S3 (see Supplementary Material). (**c**) The performance was assessed with ROC curves for individual isoform expression values (*GATA6 Em*, *GATA6 Ad*, *NKX2-1 Em*, *NKX2-1 Ad*), their respective embryonic/adult ratios (*GATA6*, *NKX2-1*), and the LC score (LC score). Exactly one random replicate per patient was selected from all samples to calculate ROC curves. See Table S4 (Supplementary Material). **d** Violin plot representing the impact of sample randomization on the performance of the LC score. Bootstrap (n = 1000) distributions of the AUC estimates. Bootstrap samples were constructed as follows: 100 random participants were sampled with replacement from the total number of 126 participants. After sampling, multiple samples for the same participant were replaced with the same number of one randomly selected EBC replicate of the respective participant before calculating AUC values. We show AUC distributions for each classifier obtained from 1000 bootstrap runs

**Fig. 2:**
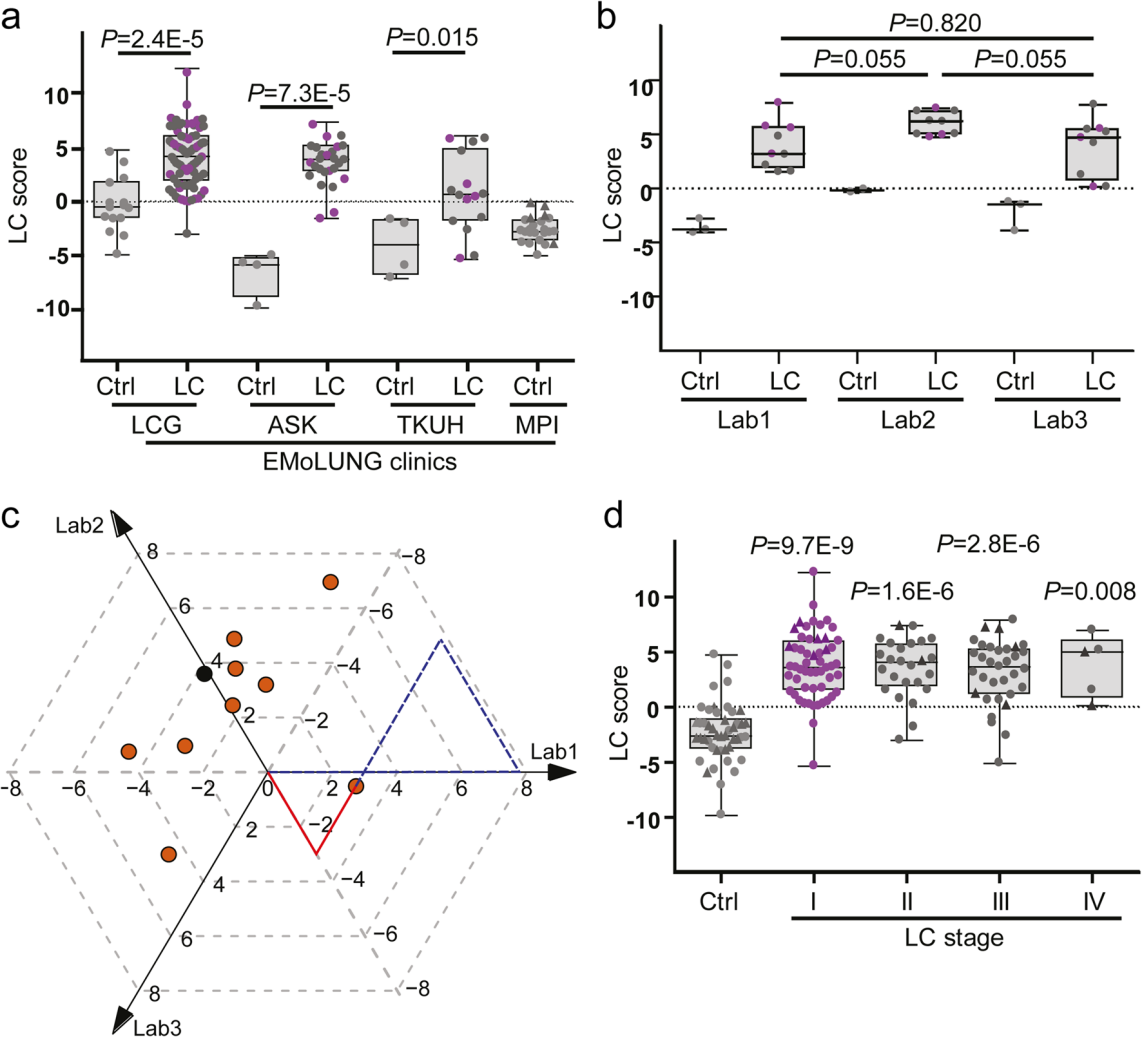
Early LC detection implementing the LC score. **a** Box plot of the LC score detected in EBCs from Ctrl and LC patients grouped based on the different clinics participating in the EMoLung study. In each box plot of this Figure, *P* values relative to Ctrl were calculated by two-sided Mann–Whitney U test. The five-number summary and the statistical test values from each box plot are shown in Table S1 (see Supplementary Material). **b** Box plot of the LC score detected in EBCs from Ctrl and LC patients grouped based on three different laboratories performing the analysis. Differences between the laboratories were not significant (Table S7 in Supplementary Material). **c** Ternary Bland–Altman plot for the inter-lab variability of the LC score. The plot shows the laboratory-specific differences of the LC score for the 10 samples that were measured in three distinct laboratories. The orange dots represent the LC samples, whereas the black dot represents the control sample. Typically, the graphical comparison between three labs is done using three Bland–Altman plots. The above representation summarizes these plots into one. The plot has three axes spanned by the vectors Lab1, Lab2, Lab3. Each sample that has been measured in triplicate (x, y, z) is mapped onto the point defined by x*Lab1 + y*Lab2 + z*Lab3. For instance, the rightmost point can be reached by several triplets, such as by the actual measurements (7.919, 4.938, 5.451) or, e.g., (0, − 2.981, − 2.468) (corresponding to the dashed blue and solid red vector paths, respectively). Triplets that map to the same point have identical y-values in all three Bland–Altman plots, and therefore are indistinguishable. The closer the points to the origin, the better the agreement between the three laboratories. The more a point is shifted away from the origin in the direction of a Lab axis, the more pronounced the deviation of the corresponding laboratory from the two others. **d** Box plot of the LC scores detected in EBCs from Ctrl and patients at LC stages I, II, III and IV. Patients were staged according to the TNM classification 8th edition. Differences between LC stages are not significant (see Table S2 in Supplementary Material). Pink circles represent LC stage I samples

The levels of adult and embryonic isoforms of *GATA6* and *NKX2-1* in each EBC were measured in triplicates and implemented for calculation of the LC score as previously described [16]. All EBCs were measured in one of three laboratories. In addition, a sample of 10 EBCs was analyzed in triplicates by different operators in the three laboratories. Statistical analysis was performed using R (4.0.2), Excel Solver and Graph Prism (v.5). Distribution of data was visualized as box plots and the corresponding five-number summaries are given in Table S1 (Supplementary Material). Two-sided Mann–Whitney U tests were calculated with one randomly picked measurement per sample to determine the statistical significance in two-group comparisons of LC scores. To provide evidence that there is no difference with respect to the LC scores between LC stages (Fig. 2d) or between LC subtypes (Fig. S3a in Supplementary Material), we applied the Mann–Whitney U test in an anticonservative way, treating replicated measurements for the same patient as independent observations. This is uncritical, because even such an anticonservative procedure did not detect any significant effect. To evaluate the differences between laboratories in Fig. 2a, b Two-sided Mann–Whitney U test was performed considering one value per donor that was randomly selected from the replicate measurements. The test values and assay IDs are provided in Tables S1, S2, S6 and S7 (see Supplementary Material). *P*-values < 0.05 were considered statistically significant. The inter-lab variability of LC scores was assessed by a ternary Bland–Altman plot and by Bland–Altman plots [29]. The performances of different LC predictors were assessed with receiver operator characteristics (ROC) analysis (R package ROCR [30]) by randomly picking exactly one replicate per donor from Lab1 in case of Fig. 1b, and one replicate per donor from all Labs in case of Fig. 1c. Sensitivities, specificities, and the respective 95% confidence intervals were calculated from [https://www2.ccrb.cuhk.edu.hk/stat/confidence%20interval/Diagnostic%20Statistic.htm] using cross tables, in which each observation was weighted by the inverse number of replicates for the selected patient.

## Results

### LC score-based classification of EBCs under clinical settings

We performed isoform-specific expression analysis by qRT–PCR after total RNA isolation from EBCs and calculated the LC scores from each patient as previously described [16] (Fig. 1a). In control EBCs (46 EBC measurements from 23 donors, Table 1 and Table S1 in Supplementary Material) the LC score was generally below 0 (the threshold above which samples are classified as LC), with a median of − 2.605 and an interquartile range of 2.770. In agreement with our previous work [16], the LC score in EBCs of LC patients was significantly higher and generally above 0 (121 EBC measurements from 103 patients; *P* = 7.3E-9), with a median of 3.717 and an interquartile range of 3.982 (Fig. 1a, Table 1 and Table S1 in Supplementary Material). These results confirm that samples with a LC score greater than zero can be classified as LC (Table S8 in Supplementary Material). To compare the performance of the LC score-based classification of the EBCs collected in EMoLung under clinical settings to the previous study under pre-clinical settings [16], we calculated ROC curves [30] (Fig. 1b and Table S3 in Supplementary Material). The area under the curve (AUC) value of the clinical study EMoLung was 0.89, whereas the AUC value of the previous pre-clinical study [16] was 0.99. Further, ROC curves for each transcript isoform, the isoform expression ratios, and for the LC score (Fig. 1c, d and Table S4 in Supplementary Material) confirmed that EBC classification achieved by the LC score was substantially better than any threshold-based classification using the expression or expression ratios of transcript isoforms from *GATA6* and *NKX2-1* alone.

### Reliable detection of stage I and II LC using the LC score

To further characterize the usefulness of the LC score under clinical conditions, EBCs for this study were prospectively collected in three different clinical centers and analyzed by different operators in three different laboratories. Sample grouping by clinical centers (Fig. 2a and Table S1 in Supplementary Material) revealed that the median LC score increased from -0.520 in control EBCs (15 measurements from 14 donors) to 4.125 (*P* = 2.4E-5) in EBCs of LC patients (80 measurements from 62 patients) in the clinical center 1 (LCG), from -5.837 in control EBCs (4 measurements from 4 donors) to 3.867 (*P* = 7.3E-5) in LC EBCs (26 measurements from 26 patients) in the clinical center 2 (ASK) and from -3.982 in control EBCs (4 measurements from 2 donors) to 0.640 (*P* = 0.015) in LC EBCs (15 measurements from 15 patients) in the clinical center 3 (TKUH).

Similarly, sample grouping by laboratories (Fig. 2b and Table S1 in Supplementary Material) revealed that the median LC score increased from -3.793 in control EBCs (3 measurements for 1 donor) to 3.173 in EBCs of LC patients (9 measurements for 9 patients) in the laboratory 1; from -0.175 in control EBCs (2 measurements for 1 donor) to 6.183 in LC EBCs (9 measurements for 9 patients) in the laboratory 2; and from -1.468 in control EBCs (3 measurements for 1 donor) to 4.689 in LC EBCs (9 measurements for 9 patients) in the laboratory 3. Interestingly, comparisons among different laboratories showed non-significant differences (Table S7 in Supplementary Material). Moreover, the reliability of the LC score-based EBC classification was monitored by a ternary Bland–Altman plot (Fig. 2c) and Bland–Altman plots [29] (Fig. S2 in Supplementary Material). In summary, the LC score proved to be highly reliable when used in different clinics and labs, corroborating its usefulness under clinical conditions.

To demonstrate that the LC score can be used for early detection of LC, samples were grouped based on TNM classification [26] (Fig. 2d, Table 1 and Table S1 in Supplementary Material). Remarkably, the median LC score increased from -2.605 in the control EBCs (46 measurements from 23 donors) to 3.604 (*P* = 9.7E-9) and 4.080 (*P* = 1.6E-6) in EBCs from patients with LC at stages I (54 measurements from 46 patients) and II (25 measurements from 23 patients), respectively. In addition, performance assessment of the LC score showed a sensitivity of 95.7% and 91.3% for stages I and II LC (Fig. 2d and Table S2 in Supplementary Material), thereby demonstrating the potential of the method for early detection of LC.

## Discussion

Performance assessment of the LC score based on the complete EBC set used in the current study revealed a sensitivity of 92.2% and specificity of 82.6% (Table S5 in Supplementary Material), compared to the sensitivity of 98.3% and specificity of 89.7% in the previous study [16]. The reduced performance of the LC score in EMoLung might be explained by increasing variance in the data due to the implementation of clinical conditions, including the participation of different centers and laboratories. Nevertheless, the statistical performance achieved by the LC score in EMoLung was still high, demonstrating the robustness of the LC score under clinical conditions. To the best of our knowledge, our LC score is the first attempt to establish a mathematical score based on the expression of embryonic- or adult-specific transcript variants. The use of isoform ratios as building blocks of the LC score make it resilient to variations that may occur at different steps of the procedure, including RNA isolation, cDNA synthesis or PCR amplification. In addition, the utility of EBCs for expression analysis has been underlined by recent studies comparing non-coding transcripts in NSCLC patients versus control donors [10, 31, 32]. Among the limitations of the present study, the LC score does not allow the distinction of LC stages (Table S2 in Supplementary Material) or NSCLC subtypes (Fig. S3 in Supplementary Material). This has already been observed in our previous study [13]. A plausible explanation for these limitations may be the sparsity of covariates included to our present LC score limiting the level of detail of its predictions. Thus, while our results are promising, we propose a larger prospective study under clinical conditions with repetitive measurements from various patients at different stages of a therapeutic approach, as this is currently ongoing within the clinical study EMoLung (Fig. S1a), and it will the scope of future reports.

Despite the limitations of EMoLung, the correct classification of Stage I-II LC samples using the LC score is encouraging. Thus, we propose that the incorporation of our method into the current protocols for patients undergoing diagnostic evaluation for pulmonary diseases characterized by hyperproliferation will be beneficial. Furthermore, complementing CT-based LC screening with our technology in high-risk populations would strengthen the screening protocols. We hypothesize that implementation of the LC score together with CT may reduce the false-positive rate of CT imaging, for example, in cases with suspicious image findings, thereby preventing individuals from unnecessary exposure to high dose of radiation or surgery.

## Conclusions

In this study, we validated in clinical settings a LC diagnostic test based on the analysis of distinct RNA isoforms expressed by the *GATA6* and *NKX2-1* gene loci detected in EBCs. LC score-based classification of EBCs achieved a sensitivity of 95.7%, 91.3% and 84.6% for LC detection at stages I-III, respectively. The LC score is an accurate and non-invasive option for early LC diagnosis and a valuable complement to LC screening procedures based on computed tomography.

### Supplementary Information

**Additional file 1**

## Data Availability

The datasets supporting the conclusions of this article are included within the article and its online Supplementary Material nformation. The data that support this study are available from the corresponding authors upon reasonable request.

## Abbreviations

AC: Adenocarcinoma
ACC: Adenoid cystic carcinoma
ASK: Asklepios Klinik Gauting GmbH
CT: Computed tomography
Ctrl: Control
CXR: Chest X-ray
EBCs: Exhaled breath condensates
IASLC: International Association for the Study of Lung Cancer
LC: Lung cancer
LCC: Large-cell carcinoma
LCG: LungenClinic Grosshansdorf GmbH
NSCLC: Non-small cell lung cancer
PET: Positron emission tomography
SCC: Squamous cell carcinoma
SOPs: Standard operating procedures
SVM: Linear support vector machine
TKUH: Thoraxklinik at Heidelberg University Hospital
