# Supplementary material for: Preliminary results from the EMoLung clinical study showing early lung cancer detection by the LC score
Source: Discov Oncol. 2023 Oct 3;14:181. doi: 10.1007/s12672-023-00799-9 (PMC10547665; doi:10.1007/s12672-023-00799-9)
Supplement: Supplementary file 1 — Additional file 1 [file 12672_2023_799_MOESM1_ESM.pdf]

Supplementary Material

Preliminary results from the EMoLung clinical study showing early lung cancer detection by the LC score

Karla Rubio, Jason M. Müller, Aditi Mehta, Iris Watermann, Till Olchers, Ina Koch, Sabine Wessels, Marc A. Schneider, Tania Araujo-Ramos, Indrabahadur Singh, Christian Kugler, Mircea Gabriel Stoleriu, Mark Kriegsmann, Martin Eichhorn, Thomas Muley, Olivia Merkel, Thomas Braun, Ole Ammerpohl, Martin Reck, Achim Tresch * and Guillermo Barreto *

* Correspondence to: guillermo.barreto@univ-lorraine.fr AND achim.tresch@uni-koeln.de

The present file contains 3 supplementary figures (Fig. S1 to S3) and 8 supplementary tables (Tables S1 to S8) with information that is relevant for this manuscript as listed below:

Fig. S1 EMoLung study flowchart.

Fig. S2 Repeatability of LC score analysis in EBCs.

Fig. S3 Repeatability of LC score analysis in EBCs.

Table S1 Five-number summaries of box plots and results of statistical significance tests.

Table S2 Validation of the LC score performance for different subsampling of the LC patients.

Table S3 Validation of the increased LC score-based performance on EBCs.

Table S4 ROC curve analysis of individual isoform expression values (*GATA6 Em, GATA6 Ad, NKX2-1 Em, NKX2-1 Ad*), their respective embryonic/adult ratios (*GATA6, NKX2-1*), and the LC score (LC score).

Table S5 Confusion Matrices.

Table S6 Bland-Altman Inter-Lab Variability.

Table S7 Wilcoxon signed rank test to compare LC scores among distinct labs.

Table S8 Values for the individual ratios of *GATA6, NKX2-1,* the LC score and the prediction of the LC score-based classifier under clinical settings.

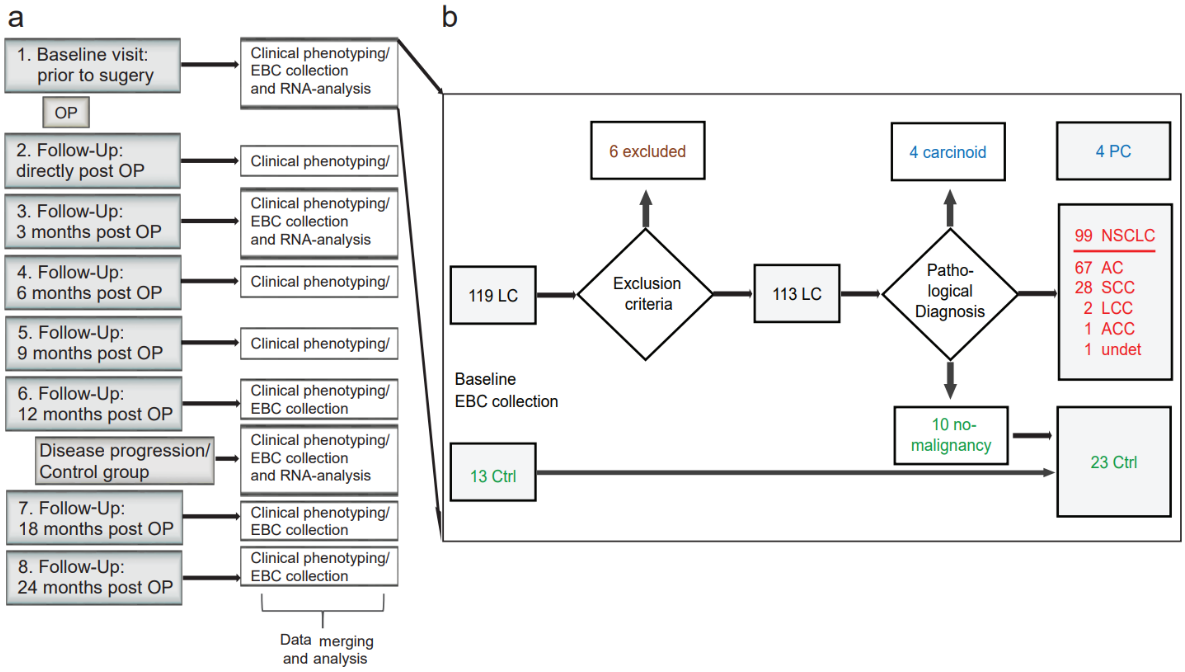

Fig. S1 (**a**) EMoLung study flowchart. In phase 1 (baseline visit: prior to surgery), EBCs were collected from prospectively enrolled controls and patients with LC. RNA-analysis of exhaled breath condensates were performed and compared with computed tomography imaging (CT). In addition, the clinical data was collected regularly in the context of the follow-up visits to establish a deep characterized patient cohort. The standard operation procedures (SOP) for the assay consisting of isoform-specific expression analysis of *GATA6* and *NKX2-1* were previously established [11] to avoid variability due to technical differences arising from instrumentation and/or platforms. For the analysis, a linear support vector machine (SVM) classifier was used to combine the Em/Ad ratios of *GATA6* and *NKX2-1* of each sample into one LC score. Detailed information of the study population can be found in Table 1. (**b**) Decision flowchart depicting the study population for the present study consisting of the base line EBCs from the clinical study EMoLung. EBCs from 119 patients were assigned to the lung cancer (LC) group, whereas EBCS from 13 donors were assigned to the control (Ctrl) group. In the LC group, 6 patients were excluded from the study based on exclusion criteria, whereas based on pathological diagnosis after operative intervention, 4 patients were diagnosed with carcinoid (PC), 99 patients with non-small cell lung cancer (NSCLC) and 10 patients with non-

malignancies. The 99 NSCLC patients were further classified as 67 adenocarcinoma (AC), 28 squamous-cell carcinoma (SCC), 2 large-cell carcinoma (LCC), 1 adenoid cystic carcinoma (ACC) and 1 was undetermined. The 10 patients with non-malignancies were reassigned to the Ctrl group, thereby resulting in EBCs from 23 donors in the Ctrl group.

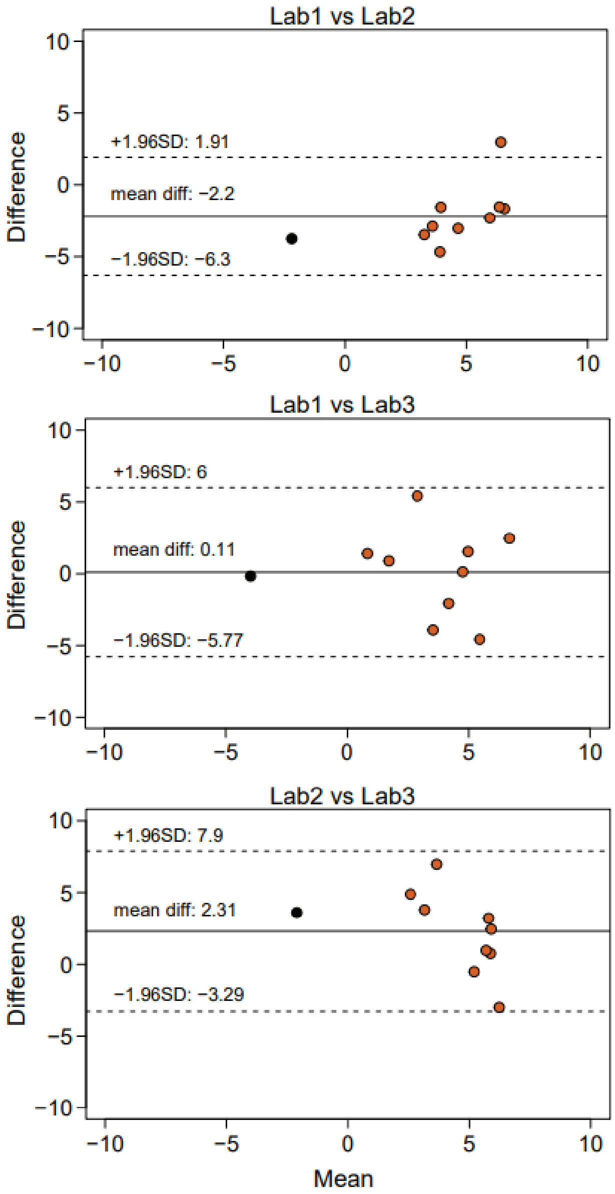

Fig. S2 Repeatability of LC score analysis in EBCs. Bland-Altman plots for the inter-lab variability of the LC scores confirmed the repeatability of LC-based analysis in EBCs. LC score-based analysis was performed by three different laboratories (Lab1, Lab2 and Lab3). Calculation of each LC score for each EBC was performed in triplicates. LC individuals were analyzed. The orange dots represent the LC samples, whereas the black dots represent the control samples. The difference between the three Tests (y-axis) was plotted against their average (x-axis). Solid line, mean of the differences; dotted lines, mean of the differences ± 1.96 * standard deviation of the difference.

a

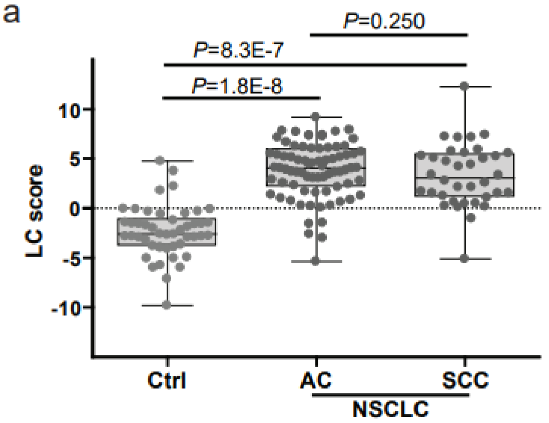

b

	LC score			
	LC subtype			
	Ctrl	AC	SCC	
N	23	67		
n	46	75		
min	-9.817	-5.329		
Q1	-3.820	2.169		
Median	-2.605	4.048		
Q3	-1.050	5.994		
max	4.740	9.176		
Mean	-2.339	3.796		
Std. Dev.	2.638	2.820		
Std. Error	0.389	0.326		
Lower 95% CI of mean	-3.122	3.147		
Upper 95% CI of mean	-1.555	4.445		
			**vs Ctrl**	**vs AC**
N			28	
n			36	
min			-5.084	
Q1			1.124	
Median			3.076	
Q3			5.491	
max			12.24	
Mean			3.310	
Std. Dev.			3.151	
Std. Error			0.525	
Lower 95% CI of mean			2.244	
Upper 95% CI of mean			4.377	
Median (for N)	-1.562	3.717	2.720	**For n**
Mann-Whitney U *Wilcoxon W* (for N)	161		79	1533
df (for N)	-		-	63
Tails (for N)	2		2	2
P Mann-Whitney U / Wilcoxon-Sum-Rank	1.8E-8 ***		8.3E-7 ***	0.250 ns

Fig. S3 Repeatability of LC score analysis in EBCs. (**a**) Box plot of the LC score detected in EBCs from control donors (Ctrl) and patients with non-small cell lung cancer (NSCLC) grouped based on histological subtypes in adenocarcinoma (AC) and squamous cell carcinoma (SCC). Patients were classified according to a pathological report. *P* values are shown for each NSCLC subtype relative to Ctrl and between NSCLC subtypes. See Table 1 and Fig. S1b (Online Resource 1). EBCs were analyzed in laboratories 1, 2 and 3. (**b**) Five-number summary and the statistical test values from the box plot in a.

Table S1 Five-number summaries of box plots and results of statistical significance tests.

	Exhaled Breath Condensates																			
	**Figure 1a**		**Figure 2b**							**Figure 2b**						**Figure 2d**				
	**LC score**		**LC score**							**LC score**						**LC score**				
			LCG		ASK		TKUH		MPI	Lab1		Lab2		Lab3		LC Stage				
	Ctrl	LC	Ctrl	LC	Ctrl	LC	Ctrl	LC	Ctrl	Ctrl	LC	Ctrl	LC	Ctrl	LC	Ctrl	I	II	III	IV
N	23	103	14	62	4	26	2	15	3	1	9	1	9	1	9	23	46			
n	46	121	15	80	4	26	4	15	23	3	9	2	9	3	9	46	54			
min	-9.817	-5.329	-4.909	-3.017	-9.817	-1.574	-7.104	-5.329	-5.002	-4.063	1.539	-0.301	4.735	-3.900	0.138	-9.817	-5.329			
Q1	-3.820	1.550	-1.562	1.828	-8.853	2.733	-6.822	-1.808	-3.627	-4.063	1.872	-0.301	4.977	-3.900	0.726	-3.820	1.530			
Median	-2.605	3.717	-0.520	4.125	-5.837	3.867	-3.982	0.640	-2.776	-3.793	3.173	-0.175	6.183	-1.468	4.689	-2.605	3.604			
Q3	-1.050	5.532	1.813	6.041	-5.180	5.152	-1.698	4.838	-1.706	-2.776	5.672	-0.050	7.137	-1.201	5.478	-1.050	6.016			
max	4.740	12.241	4.740	12.241	-5.002	7.305	-1.601	6.044	-0.050	-2.776	7.919	-0.050	7.407	-1.201	7.744	4.74	12.241			
Mean	-2.339	3.570	-0.304	4.084	-6.623	3.615	-4.167	0.745	-2.602	-3.544	3.965	-0.175	5.985	-2.189	3.822	-2.339	3.746			
Std. Dev.	2.638	2.891	2.584	2.679	2.168	2.031	2.782	3.701	1.205	0.679	2.189	0.178	1.075	1.487	2.670	2.638	3.024			
Std. Error	0.389	0.263	0.667	0.300	1.084	0.398	1.391	0.956	0.251	0.392	0.730	0.126	0.358	0.859	0.890	0.389	0.412			
Lower 95% CI of mean	-3.122	3.049	-1.736	3.488	-10.072	2.795	-8.594	-1.304	-3.123	-5.230	2.282	-1.774	5.159	-5.883	1.770	-3.122	2.921			
Upper 95% CI of mean	-1.555	4.090	1.127	4.681	-3.174	4.435	0.259	2.795	-2.081	-1.858	5.647	1.424	6.812	1.505	5.875	-1.555	4.572			
																		**vs Ctrl**		
N																		23	26	3
n																		25	33	5
Min																		-3.017	-5.084	0.138
Q1																		1.858	1.173	0.838
Median																		4.080	3.672	5.017
Q3																		5.721	5.268	6.078
Max																		7.407	7.919	6.981
Mean																		3.501	3.122	3.770
Std. Dev.																		2.639	2.948	2.829
Std. Error																		0.528	0.513	1.265
Lower 95% CI of mean																		2.411	2.077	0.257
Upper 95% CI of mean																		4.590	4.167	7.282
Median (for N)	-1.562	3.426	-0.430	3.54	-5.837	3.867	-6.539	0.64	-	**For n**						-1.562	3.157	3.717	3.284	5.175
Mann-Whitney U / Wilcoxon W (for N)	268		118		0		0		-	0		0		0		145		86	132	8
df (for N)	-		-		-		-		-	9		8		6		-		-	-	-
Tails (for N)	2		2		2		2		-	2		2		2		2		2	2	2
P Mann-Whitney U / Wilcoxon-Rank-Sum	7.3E-9		2.4E-5		7.3E-5		0.015		-							9.7E-9		1.6E-6	5E-6	0.008
	***		***		***		*		-	**		*		**		***		***	***	**

Five-number summaries of the box plots presented in the indicated Figures. N refers to the number of participants; n refers to the number of measurements in the set. Total N value in control groups (Ctrl) is 23; total n value is 46. Total N value in lung cancer group (LC) is 103; total n value is 121. Minimum (min) and maximum (max) values; first

(Q1) and third (Q3) quartile. Two-sided Mann-Whitney U tests were performed to determine the statistical significance of the difference between Ctrl and LC samples. In addition, to assess the differences between the LC stages in the Figure 2d, the Mann-Whitney U test was anticonservative, since replicate measurements for the same patient were used as independent observations to evaluate technical variability of the method (see Supplementary Table 2). Degree of freedom (df) and *P*-values are presented. Samples collected in Grosshansdorf (LCG), Munich (ASK), Heidelberg (TKUH) and Bad Nauheim (MPI) in Germany.

Table S2 Validation of the LC score performance for different subsampling of the LC patients.

**LC stage 1-2**	**Disease**	**No Disease**	
LC	65	4	
Ctrl	4	19	
	**Point Estimate**	**lower C.I.**	**upper C.I.**
Sensitivity	0.94203	0.88689	0.99717
Specificity	0.82609	0.67118	0.98099
**LC stage 1**	**Disease**	**No Disease**	
LC	44	2	
Ctrl	4	19	
	**Point Estimate**	**lower C.I.**	**upper C.I.**
Sensitivity	0.95652	0.89759	1.01545
Specificity	0.82609	0.67118	0.98099
**LC stage 2**	**Disease**	**No Disease**	
LC	21	2	
Ctrl	4	19	
	**Point Estimate**	**lower C.I.**	**upper C.I.**
Sensitivity	0.91304	0.79789	1.0282
Specificity	0.82609	0.67118	0.98099
**LC stage 3**	**Disease**	**No Disease**	
LC	22	4	
Ctrl	4	19	
	**Point Estimate**	**lower C.I.**	**upper C.I.**
Sensitivity	0.84615	0.70747	0.98484
Specificity	0.82609	0.67118	0.98099
**LC stage 4**	**Disease**	**No Disease**	
LC	3	0	
Ctrl	4	19	
	**Point Estimate**	**lower C.I.**	**upper C.I.**
Sensitivity	1	1	1
Specificity	0.82609	0.67118	0.98099

Comparison LC score of LC Stage P-value (Wilcoxon-Rank-Sum/Mann-Whitney-U)	
1 vs 2	0.9202
1 vs 3	0.4449
1 vs 4	0.9674
2 vs 3	0.4872
2 vs 4	0.957
3 vs 4	0.6821

C.I. calculated with https://www2.ccrb.cuhk.edu.hk/stat/confidence%20interval/Diagnostic%20Statistic.htm
Replicate measurements for the same person were weighted by the inverse number of replicates when constructing the confusion matrix, respectively
True positive rate (TPR), true negative rate (TNR), positive predicted value (PPV) and negative predicted value (NPV). 1-IV, LC stages compared to Ctrl.
Wilcoxon-Rank-Sum/Mann-Whitney-U Test is anti-conservative, since the groups comprise multiple samples for the same patient.

Table S3: Validation of LC score-based performance on EBCs under clinical settings.

	Min	5%	25%	Median	75%	95%	Max	Mean	Std. Dev.	Count
Ctrl	<0.001	<0.001	<0.001	<0.001	<0.001	3.61	4.74	<0.001	3.57	23
LC	<0.001	<0.001	1.53	3.43	5.3	7.38	12.2	3.34	2.93	103

Cut-point results for target Sensitivity (S)

Target Se	Cut-point	Sensitivity	S Lower 95% CL	S Upper 95% CL	Specificity	Sp Lower 95% CL	Sp Upper 95% CL
0.999	-5.329	1	0.964	1	0.217	0.097	0.419
0.995	-5.329	1	0.964	1	0.217	0.097	0.419
0.99	-5.084	0.99	0.947	0.998	0.217	0.097	0.419
0.98	-3.017	0.981	0.932	0.995	0.348	0.188	0.551
0.95	-1.574	0.951	0.891	0.979	0.478	0.292	0.67
0.9	0.239	0.903	0.83	0.946	0.826	0.629	0.93
0.8	1.043	0.806	0.719	0.871	0.826	0.629	0.93

Cut-point results for target Specificity (Sp)

Target Sp	Cut-point	Specificity	Sp Lower 95% CL	Sp Upper 95% CL	Sensitivity	S Lower 95% CL	S Upper 95% CL
0.999	4.823	1	0.857	1	0.33	0.247	0.426
0.995	4.823	1	0.857	1	0.33	0.247	0.426
0.99	4.823	1	0.857	1	0.33	0.247	0.426
0.98	4.823	1	0.857	1	0.33	0.247	0.426
0.95	3.844	0.957	0.79	0.992	0.437	0.345	0.533
0.9	2.343	0.913	0.732	0.976	0.66	0.564	0.744
0.8	0.034	0.826	0.629	0.93	0.922	0.854	0.96

**Area under curve (AUC)**	0.887
**95% CI for AUC**	0.807-0.967

Table S4 Validation of the increased LC score-based performance on EBCs.

Data frame with all samples, including replicates and measures in labs 1-3 for some patients				
**Name**	**AUC**	**Optimal Cut-Off**	**Optimal Specificity**	**Optimal Sensitivity**
GATA6em	0.62756019	-36.2558047	0.6086957	0.6280992
GATA6ad	0.72044556	35.7492383	0.7391304	0.7024793
NKX2-1em	0.66924183	-38.1176402	0.5652174	0.7355372
NKX2-1ad	0.80632411	36.9169769	0.7608696	0.7355372
GATA6	0.8420769	0.54	0.7173913	0.8677686
NKX2-1	0.86094143	0.651	0.8695652	0.7520661
LC Score	0.93262666	0.034	0.9130435	0.9338843
**Data frame with one replicate per patient from lab 1**				
**Name**	**AUC**	**Optimal Cut-Off**	**Optimal Specificity**	**Optimal Sensitivity**
GATA6em	0.65386239	-36.2558047	0.6521739	0.631068
GATA6ad	0.64710848	35.6254323	0.6521739	0.7572816
NKX2-1em	0.74166315	-38.5297877	0.5652174	0.8058252
NKX2-1ad	0.7982271	36.8906835	0.7826087	0.7378641
GATA6	0.75749261	2.387	0.7826087	0.592233
NKX2-1	0.87083157	0.548	0.8695652	0.7669903
LC Score	0.8868721	0.034	0.826087	0.9223301

Optimal cut-off were calculated by getting the closest point to TPR=1 and FPR=0.
“Optimal” in the sense it weights both sensitivity and specificity equally.

Table S5 Confusion Matrices.

LC Score (Confusion Matrix, samples from lab 1 with weighted replicates for the same patient)			
	**Disease**	**No Disease**	
**LC**	95	8	
**Ctrl**	4	19	
	**Point Estimate**	**lower C.I.**	**upper C.I.**
**Sensitivity**	0.92233	0.87064	0.97402
**Specificity**	0.82609	0.67118	0.98099
**LC Score (Confusion Matrix, samples from lab1 and one replicate per patient)**			
	**Disease**	**No Disease**	
**LC**	95	8	
**Ctrl**	4	19	
	**Point Estimate**	**lower C.I.**	**upper C.I.**
**Sensitivity**	0.92233	0.87064	0.97402
**Specificity**	0.82609	0.67118	0.98099

C.I. calculated with https://www2.ccrb.cuhk.edu.hk/stat/confidence%20interval/Diagnostic%20Statistic.htm
Replicate measurements for the same person were weighted by the inverse number of replicates when constructing the confusion matrix, respectively

Table S6 Bland-Altman Inter-Lab Variability.

PatientID	AssayID Lab1	AssayID Lab2	AssayID Lab3	LC Score Lab1	LC Score Lab2	LC Score Lab3
**Ctrl11**	17	161	166	-4.063	-0.3012216	-3.899793
**LC101**	128	156	162	4.823	7.1344983	4.688677
**LC105**	132	159	165	3.14	6.1825654	5.2197
**LC111**	138	157	163	5.744	7.4072995	4.202976
**LC53**	81	153	169	1.579	6.2564	5.5041
**LC84**	112	154	170	7.919	4.9376829	5.451102
**LC89**	117	158	164	5.599	7.1387551	0.182443
**LC92**	120	151	167	3.173	4.734669	7.7444
**LC95**	122	152	168	1.539	5.0169168	0.137578
**LC97**	124	155	171	2.165	5.060618	1.2701
**Bland-Altman Plot**						
**Comparison**	**Mean Diff**	**+1.96SD**	**-1.96SD**			
**Lab1 vs Lab2**	-2.1950180	1.905563	-6.2956			
**Lab1 vs Lab3**	0.1116717	5.995969	-5.772625			
**Lab2 vs Lab3**	2.30669	7.903002	-3.289622			

Table S7 Wilcoxon signed rank test to compare LC scores among distinct labs.

Comparison (LC vs LC)	*P*-value (Wilcoxon signed rank exact test)	V-value
**Lab1 vs Lab2**	0.05469	6
**Lab1 vs Lab3**	0.8203	25
**Lab2 vs Lab3**	0.05469	39
**Lab**	**LC Median**	**LC sample size**
**Lab1**	3.173	9
**Lab2**	6.182565	9
**Lab3**	4.688677	9

Table S8 Values for the individual ratios of *GATA6*, *NKX2-1*, the LC score and the prediction of the LC score-based classifier under clinical settings.

Assay ID	Patient ID	Clinic	Ratio *GATA6*	Ratio *NKX2-1*	LC score	Pathology	LC subtype	Disease state	Predicted correct	Lab
1	Ctrl1	TKUH	0.032	0.019	-7.104	Control	Control	Control	Yes	1
2	Ctrl2	TKUH	0.399	0.203	-1.601	Control	Control	Control	Yes	1
3	Ctrl1	TKUH	0.252	0.218	-1.990	Control	Control	Control	Yes	1
4	Ctrl2	TKUH	0.323	0.007	-5.974	Control	Control	Control	Yes	1
5	Ctrl3	ASK	0.070	0.055	-5.002	Control	Control	Control	Yes	1
6	Ctrl4	ASK	0.014	0.120	-5.713	Control	Control	Control	Yes	1
7	Ctrl5	ASK	0.023	0.066	-5.960	Control	Control	Control	Yes	1
8	Ctrl6	ASK	0.001	0.037	-9.817	Control	Control	Control	Yes	1
9	Ctrl7	LCG	0.148	0.125	-3.222	Control	Control	Control	Yes	1
10	Ctrl7	LCG	0.921	0.117	-1.423	Control	Control	Control	Yes	1
11	Ctrl8	LCG	3.213	0.121	-0.086	Control	Control	Control	Yes	1
12	Ctrl9	LCG	0.515	0.170	-1.562	Control	Control	Control	Yes	1
13	Ctrl10	LCG	0.462	0.067	-2.827	Control	Control	Control	Yes	1
14	Ctrl11	MPI	0.087	0.123	-3.793	Control	Control	Control	Yes	1
15	Ctrl12	MPI	0.089	0.045	-5.002	Control	Control	Control	Yes	1
16	Ctrl11	MPI	0.123	0.210	-2.776	Control	Control	Control	Yes	1
17	Ctrl11	MPI	0.067	0.122	-4.063	Control	Control	Control	Yes	1
18	Ctrl12	MPI	0.161	0.159	-2.842	Control	Control	Control	Yes	1
19	Ctrl13	MPI	0.288	0.161	-2.221	Control	Control	Control	Yes	1
20	Ctrl13	MPI	0.074	0.288	-2.912	Control	Control	Control	Yes	1
21	Ctrl13	MPI	0.045	0.242	-3.627	Control	Control	Control	Yes	1
22	Ctrl13	MPI	0.047	0.970	-1.869	Control	Control	Control	Yes	1
23	Ctrl13	MPI	0.186	0.398	-1.559	Control	Control	Control	Yes	1
24	Ctrl13	MPI	0.010	1.897	-2.612	Control	Control	Control	Yes	1
25	Ctrl13	MPI	0.065	0.317	-2.929	Control	Control	Control	Yes	1
26	Ctrl13	MPI	0.057	0.346	-2.949	Control	Control	Control	Yes	1
27	Ctrl13	MPI	0.045	0.465	-2.836	Control	Control	Control	Yes	1
28	Ctrl13	MPI	0.157	0.407	-1.706	Control	Control	Control	Yes	1
29	Ctrl13	MPI	0.916	0.045	-2.598	Control	Control	Control	Yes	1
30	Ctrl13	MPI	0.045	0.522	-2.690	Control	Control	Control	Yes	1
31	Ctrl13	MPI	0.231	0.048	-3.940	Control	Control	Control	Yes	1

**32**	**LC71**	**LCG**	**0.741**	**0.291**	**-0.520**	**No malignancy**	**Control**	**Control**	**Yes**	**1**
**33**	**LC75**	**LCG**	**3.416**	**5.766**	**4.740**	**No malignancy**	**Control**	**Control**	**No**	**1**
**34**	**LC81**	**LCG**	**2.274**	**0.107**	**-0.598**	**No malignancy**	**Control**	**Control**	**Yes**	**1**
**35**	**LC85**	**LCG**	**17.036**	**0.024**	**-0.340**	**No malignancy**	**Control**	**Control**	**Yes**	**1**
**36**	**LC86**	**LCG**	**2.316**	**0.158**	**-0.095**	**No malignancy**	**Control**	**Control**	**Yes**	**1**
**37**	**LC100**	**LCG**	**1.677**	**0.065**	**-1.527**	**No malignancy**	**Control**	**Control**	**Yes**	**1**
**38**	**LC104**	**LCG**	**0.006**	**0.478**	**-4.909**	**No malignancy**	**Control**	**Control**	**Yes**	**1**
**39**	**LC106**	**LCG**	**2.128**	**0.798**	**1.813**	**No malignancy**	**Control**	**Control**	**No**	**1**
**40**	**LC108**	**LCG**	**5.034**	**0.544**	**2.229**	**No malignancy**	**Control**	**Control**	**No**	**1**
**41**	**LC114**	**LCG**	**5.048**	**1.880**	**3.760**	**No malignancy**	**Control**	**Control**	**No**	**1**
**42**	**Ctrl11**	**MPI**	**0.071**	**3.029**	**-0.050**	**Control**	**Control**	**Control**	**Yes**	**2**
**43**	**Ctrl11**	**MPI**	**0.395**	**0.589**	**-0.301**	**Control**	**Control**	**Control**	**Yes**	**2**
**44**	**Ctrl11**	**MPI**	**0.057**	**0.161**	**-3.900**	**Control**	**Control**	**Control**	**Yes**	**3**
**45**	**Ctrl11**	**MPI**	**0.485**	**0.239**	**-1.201**	**Control**	**Control**	**Control**	**Yes**	**3**
**46**	**Ctrl11**	**MPI**	**1.679**	**0.068**	**-1.468**	**Control**	**Control**	**Control**	**Yes**	**3**
**47**	**LC1**	**TKUH**	**2.148**	**0.711**	**1.678**	**Malignancy**	**NSCLC-AC**	**LC**	**Yes**	**1**
**48**	**LC2**	**TKUH**	**0.222**	**0.147**	**-2.607**	**Malignancy**	**NSCLC-AC**	**LC**	**No**	**1**
**49**	**LC3**	**TKUH**	**0.704**	**0.778**	**0.640**	**Malignancy**	**NSCLC-AC**	**LC**	**Yes**	**1**
**50**	**LC4**	**TKUH**	**1.337**	**0.498**	**0.752**	**Malignancy**	**NSCLC-AC**	**LC**	**Yes**	**1**
**51**	**LC5**	**TKUH**	**0.418**	**0.217**	**-1.473**	**Malignancy**	**NSCLC-AC**	**LC**	**No**	**1**
**52**	**LC6**	**TKUH**	**6.138**	**9.760**	**5.994**	**Malignancy**	**NSCLC-AC**	**LC**	**Yes**	**1**
**53**	**LC7**	**TKUH**	**0.458**	**0.806**	**0.239**	**Malignancy**	**NSCLC-AC**	**LC**	**Yes**	**1**
**54**	**LC8**	**TKUH**	**2.929**	**7.105**	**4.838**	**Malignancy**	**NSCLC-AC**	**LC**	**Yes**	**1**
**55**	**LC9**	**TKUH**	**3.264**	**13.022**	**5.698**	**Malignancy**	**NSCLC-AC**	**LC**	**Yes**	**1**
**56**	**LC10**	**TKUH**	**6.071**	**10.263**	**6.044**	**Malignancy**	**NSCLC-AC**	**LC**	**Yes**	**1**
**57**	**LC11**	**TKUH**	**0.502**	**0.142**	**-1.808**	**Malignancy**	**NSCLC-LCC**	**LC**	**No**	**1**
**58**	**LC12**	**TKUH**	**0.877**	**0.921**	**1.075**	**Malignancy**	**NSCLC-SCC**	**LC**	**Yes**	**1**
**59**	**LC13**	**TKUH**	**0.067**	**0.044**	**-5.329**	**Malignancy**	**NSCLC-AC**	**LC**	**No**	**1**
**60**	**LC14**	**TKUH**	**0.051**	**0.067**	**-5.084**	**Malignancy**	**NSCLC-SCC**	**LC**	**No**	**1**
**61**	**LC15**	**TKUH**	**1.474**	**0.382**	**0.524**	**Malignancy**	**NSCLC-SCC**	**LC**	**Yes**	**1**
**62**	**LC16**	**ASK**	**2.875**	**3.704**	**4.016**	**Malignancy**	**NSCLC-AC**	**LC**	**Yes**	**1**

**63**	**LC17**	**ASK**	**6.983**	**3.074**	**4.701**	**Malignancy**	**NSCLC-SCC**	**LC**	**Yes**	**1**
**64**	**LC18**	**ASK**	**0.057**	**1.056**	**-1.574**	**Malignancy**	**NSCLC-AC**	**LC**	**No**	**1**
**65**	**LC20**	**ASK**	**3.227**	**20.654**	**6.255**	**Malignancy**	**NSCLC-AC**	**LC**	**Yes**	**1**
**66**	**LC21**	**ASK**	**1.086**	**0.143**	**-1.003**	**Malignancy**	**NSCLC-SCC**	**LC**	**No**	**1**
**67**	**LC22**	**ASK**	**3.920**	**3.992**	**4.428**	**Malignancy**	**NSCLC-AC**	**LC**	**Yes**	**1**
**68**	**LC23**	**ASK**	**5.142**	**1.676**	**3.638**	**Malignancy**	**NSCLC-AC**	**LC**	**Yes**	**1**
**69**	**LC24**	**ASK**	**9.770**	**0.548**	**2.920**	**Malignancy**	**NSCLC-AC**	**LC**	**Yes**	**1**
**70**	**LC25**	**ASK**	**0.189**	**4.263**	**1.384**	**Malignancy**	**NSCLC-AC**	**LC**	**Yes**	**1**
**71**	**LC26**	**ASK**	**0.756**	**2.502**	**2.154**	**Malignancy**	**NSCLC-SCC**	**LC**	**Yes**	**1**
**72**	**LC27**	**ASK**	**64.861**	**0.135**	**3.141**	**Malignancy**	**NSCLC-AC**	**LC**	**Yes**	**1**
**73**	**LC28**	**ASK**	**16.938**	**0.319**	**2.821**	**Malignancy**	**NSCLC-SCC**	**LC**	**Yes**	**1**
**74**	**LC29**	**ASK**	**4.932**	**3.167**	**4.380**	**Malignancy**	**NSCLC-SCC**	**LC**	**Yes**	**1**
**75**	**LC30**	**ASK**	**4.186**	**2.819**	**4.067**	**Malignancy**	**NSCLC-AC**	**LC**	**Yes**	**1**
**76**	**LC31**	**ASK**	**17.475**	**2.295**	**5.287**	**Malignancy**	**NSCLC-SCC**	**LC**	**Yes**	**1**
**77**	**LC32**	**ASK**	**27.059**	**3.226**	**6.158**	**Malignancy**	**NSCLC-AC**	**LC**	**Yes**	**1**
**78**	**LC33**	**ASK**	**0.977**	**2.602**	**2.467**	**Malignancy**	**NSCLC-AC**	**LC**	**Yes**	**1**
**79**	**LC34**	**ASK**	**20.615**	**1.837**	**5.183**	**Malignancy**	**NSCLC-AC**	**LC**	**Yes**	**1**
**80**	**LC35**	**ASK**	**13.284**	**1.085**	**4.080**	**Malignancy**	**NSCLC-AC**	**LC**	**Yes**	**1**
**81**	**LC36**	**ASK**	**7.796**	**4.617**	**5.317**	**Malignancy**	**NSCLC-AC**	**LC**	**Yes**	**1**
**82**	**LC37**	**ASK**	**1.142**	**6.291**	**3.717**	**Malignancy**	**NSCLC-AC**	**LC**	**Yes**	**1**
**83**	**LC38**	**ASK**	**3.499**	**1.859**	**3.369**	**Malignancy**	**NSCLC-SCC**	**LC**	**Yes**	**1**
**84**	**LC39**	**ASK**	**5.845**	**0.957**	**3.079**	**Malignancy**	**NSCLC-AC**	**LC**	**Yes**	**1**
**85**	**LC41**	**ASK**	**37.674**	**6.194**	**7.305**	**Malignancy**	**NSCLC-AC**	**LC**	**Yes**	**1**
**86**	**LC42**	**ASK**	**5.370**	**0.300**	**1.561**	**Malignancy**	**NSCLC-SCC**	**LC**	**Yes**	**1**
**87**	**LC43**	**ASK**	**7.588**	**4.094**	**5.141**	**Malignancy**	**NSCLC-AC**	**LC**	**Yes**	**1**
**88**	**LC44**	**LCG**	**225.905**	**75.742**	**12.241**	**Malignancy**	**NSCLC-SCC**	**LC**	**Yes**	**1**
**89**	**LC46**	**LCG**	**0.185**	**0.123**	**-3.017**	**Malignancy**	**NSCLC-AC**	**LC**	**No**	**1**
**90**	**LC49**	**LCG**	**2.764**	**52.423**	**7.245**	**Malignancy**	**NSCLC-SCC**	**LC**	**Yes**	**1**
**91**	**LC50**	**LCG**	**176.357**	**0.108**	**3.903**	**Malignancy**	**NSCLC-AC**	**LC**	**Yes**	**1**
**92**	**LC51**	**LCG**	**166.752**	**0.040**	**2.618**	**Malignancy**	**NSCLC-SCC**	**LC**	**Yes**	**1**
**93**	**LC52**	**LCG**	**0.778**	**3.487**	**2.593**	**Malignancy**	**NSCLC-AC**	**LC**	**Yes**	**1**
**94**	**LC53**	**LCG**	**5.465**	**0.300**	**1.579**	**Malignancy**	**NSCLC-AC**	**LC**	**Yes**	**1**
**95**	**LC55**	**LCG**	**0.215**	**2.099**	**0.641**	**Malignancy**	**NSCLC-SCC**	**LC**	**Yes**	**1**
**96**	**LC56**	**LCG**	**7.805**	**0.651**	**2.901**	**Malignancy**	**NSCLC-ACC**	**LC**	**Yes**	**1**

**97**	**LC58**	**LCG**	**0.778**	**0.543**	**0.299**	**Malignancy**	**NSCLC-AC**	**LC**	**Yes**	**1**
**98**	**LC59**	**LCG**	**11.412**	**4.085**	**5.559**	**Malignancy**	**NSCLC-SCC**	**LC**	**Yes**	**1**
**99**	**LC60**	**LCG**	**1.506**	**2.340**	**2.783**	**Malignancy**	**NSCLC-AC**	**LC**	**Yes**	**1**
**100**	**LC61**	**LCG**	**0.635**	**2.015**	**1.707**	**Malignancy**	**NSCLC-AC**	**LC**	**Yes**	**1**
**101**	**LC62**	**LCG**	**11.064**	**0.058**	**0.288**	**Malignancy**	**NSCLC-AC**	**LC**	**Yes**	**1**
**102**	**LC63**	**LCG**	**2.733**	**0.425**	**1.293**	**Malignancy**	**NSCLC-AC**	**LC**	**Yes**	**1**
**103**	**LC64**	**LCG**	**4.851**	**1.372**	**3.331**	**Malignancy**	**NSCLC-SCC**	**LC**	**Yes**	**1**
**104**	**LC65**	**LCG**	**15.515**	**1.086**	**4.241**	**Malignancy**	**NSCLC-AC**	**LC**	**Yes**	**1**
**105**	**LC66**	**LCG**	**0.735**	**0.478**	**0.084**	**Malignancy**	**NSCLC-AC**	**LC**	**Yes**	**1**
**106**	**LC67**	**LCG**	**2.397**	**3.179**	**3.640**	**Malignancy**	**NSCLC-AC**	**LC**	**Yes**	**1**
**107**	**LC68**	**LCG**	**0.025**	**7.826**	**0.034**	**Malignancy**	**PC**	**LC**	**Yes**	**1**
**108**	**LC69**	**LCG**	**4.471**	**39.054**	**7.378**	**Malignancy**	**NSCLC-AC**	**LC**	**Yes**	**1**
**109**	**LC70**	**LCG**	**1.448**	**4.365**	**3.511**	**Malignancy**	**NSCLC-LCC**	**LC**	**Yes**	**1**
**110**	**LC72**	**LCG**	**18.277**	**15.835**	**7.716**	**Malignancy**	**NSCLC-AC**	**LC**	**Yes**	**1**
**111**	**LC73**	**LCG**	**7.934**	**13.641**	**6.672**	**Malignancy**	**NSCLC-AC**	**LC**	**Yes**	**1**
**112**	**LC74**	**LCG**	**0.540**	**80.038**	**6.082**	**Malignancy**	**NSCLC-AC**	**LC**	**Yes**	**1**
**113**	**LC76**	**LCG**	**78.898**	**15.217**	**9.176**	**Malignancy**	**NSCLC-AC**	**LC**	**Yes**	**1**
**114**	**LC77**	**LCG**	**40.096**	**5.740**	**7.275**	**Malignancy**	**NSCLC-AC**	**LC**	**Yes**	**1**
**115**	**LC78**	**LCG**	**0.784**	**9.542**	**3.844**	**Malignancy**	**NSCLC-AC**	**LC**	**Yes**	**1**
**116**	**LC79**	**LCG**	**24.442**	**6.842**	**6.981**	**Malignancy**	**NSCLC-AC**	**LC**	**Yes**	**1**
**117**	**LC80**	**LCG**	**1.123**	**5.657**	**3.569**	**Malignancy**	**NSCLC-AC**	**LC**	**Yes**	**1**
**118**	**LC82**	**LCG**	**0.802**	**27.559**	**5.175**	**Malignancy**	**NSCLC-AC**	**LC**	**Yes**	**1**
**119**	**LC83**	**LCG**	**0.631**	**74.639**	**6.157**	**Malignancy**	**NSCLC-AC**	**LC**	**Yes**	**1**
**120**	**LC84**	**LCG**	**75.491**	**5.697**	**7.919**	**Malignancy**	**NSCLC-AC**	**LC**	**Yes**	**1**
**121**	**LC87**	**LCG**	**9.919**	**6.144**	**5.918**	**Malignancy**	**NSCLC-SCC**	**LC**	**Yes**	**1**
**122**	**LC88**	**LCG**	**2.392**	**12.755**	**5.352**	**Malignancy**	**NSCLC-AC**	**LC**	**Yes**	**1**
**123**	**LC89**	**LCG**	**14.054**	**3.545**	**5.599**	**Malignancy**	**NSCLC-SCC**	**LC**	**Yes**	**1**
**124**	**LC90**	**LCG**	**0.119**	**2.545**	**0.270**	**Malignancy**	**NSCLC-SCC**	**LC**	**Yes**	**1**
**125**	**LC91**	**LCG**	**1.915**	**0.402**	**0.857**	**Malignancy**	**NSCLC-AC**	**LC**	**Yes**	**1**
**126**	**LC92**	**LCG**	**1.662**	**2.958**	**3.173**	**Malignancy**	**NSCLC-AC**	**LC**	**Yes**	**1**
**127**	**LC94**	**LCG**	**0.347**	**4.860**	**2.169**	**Malignancy**	**NSCLC-AC**	**LC**	**Yes**	**1**
**128**	**LC95**	**LCG**	**1.657**	**0.788**	**1.539**	**Malignancy**	**NSCLC-SCC**	**LC**	**Yes**	**1**
**129**	**LC96**	**LCG**	**0.486**	**1.599**	**1.147**	**Malignancy**	**PC**	**LC**	**Yes**	**1**
**130**	**LC97**	**LCG**	**1.370**	**1.535**	**2.165**	**Malignancy**	**NSCLC-SCC**	**LC**	**Yes**	**1**
**131**	**LC98**	**LCG**	**5.647**	**5.646**	**5.232**	**Malignancy**	**NSCLC-SCC**	**LC**	**Yes**	**1**
**132**	**LC99**	**LCG**	**3.538**	**1.930**	**3.426**	**Malignancy**	**NSCLC-AC**	**LC**	**Yes**	**1**

**133**	**LC101**	**LCG**	**4.703**	**4.718**	**4.823**	**Malignancy**	**NSCLC-AC**	**LC**	**Yes**	**1**
**134**	**LC102**	**LCG**	**2.387**	**0.245**	**0.477**	**Malignancy**	**NSCLC-SCC**	**LC**	**Yes**	**1**
**135**	**LC105**	**LCG**	**0.334**	**11.024**	**3.140**	**Malignancy**	**PC**	**LC**	**Yes**	**1**
**136**	**LC107**	**LCG**	**10.724**	**2.875**	**5.062**	**Malignancy**	**Undetermined**	**LC**	**Yes**	**1**
**137**	**LC109**	**LCG**	**7.459**	**0.259**	**1.716**	**Malignancy**	**NSCLC-SCC**	**LC**	**Yes**	**1**
**138**	**LC110**	**LCG**	**39.980**	**0.055**	**1.530**	**Malignancy**	**NSCLC-SCC**	**LC**	**Yes**	**1**
**139**	**LC111**	**LCG**	**10.045**	**5.280**	**5.744**	**Malignancy**	**NSCLC-SCC**	**LC**	**Yes**	**1**
**140**	**LC113**	**LCG**	**5.301**	**3.382**	**4.535**	**Malignancy**	**NSCLC-AC**	**LC**	**Yes**	**1**
**141**	**LC116**	**LCG**	**3.290**	**3.396**	**4.048**	**Malignancy**	**NSCLC-AC**	**LC**	**Yes**	**1**
**142**	**LC118**	**LCG**	**85.927**	**2.746**	**7.151**	**Malignancy**	**NSCLC-SCC**	**LC**	**Yes**	**1**
**143**	**LC119**	**LCG**	**2.198**	**3.507**	**3.672**	**Malignancy**	**NSCLC-AC**	**LC**	**Yes**	**1**
**144**	**LC120**	**LCG**	**3.219**	**0.868**	**2.343**	**Malignancy**	**NSCLC-AC**	**LC**	**Yes**	**1**
**145**	**LC121**	**LCG**	**2.073**	**0.437**	**1.043**	**Malignancy**	**NSCLC-AC**	**LC**	**Yes**	**1**
**146**	**LC122**	**LCG**	**3.366**	**1.531**	**3.089**	**Malignancy**	**NSCLC-AC**	**LC**	**Yes**	**1**
**147**	**LC123**	**LCG**	**0.922**	**3.226**	**2.673**	**Malignancy**	**PC**	**LC**	**Yes**	**1**
**148**	**LC124**	**LCG**	**69.979**	**5.574**	**7.813**	**Malignancy**	**NSCLC-AC**	**LC**	**Yes**	**1**
**149**	**LC125**	**LCG**	**28.768**	**8.152**	**7.365**	**Malignancy**	**NSCLC-AC**	**LC**	**Yes**	**1**
**150**	**LC92**	**LCG**	**0.776**	**19.817**	**4.735**	**Malignancy**	**NSCLC-AC**	**LC**	**Yes**	**2**
**151**	**LC95**	**LCG**	**0.817**	**23.858**	**5.017**	**Malignancy**	**NSCLC-SCC**	**LC**	**Yes**	**2**
**152**	**LC53**	**LCG**	**0.800**	**66.347**	**6.256**	**Malignancy**	**NSCLC-AC**	**LC**	**Yes**	**2**
**153**	**LC84**	**LCG**	**0.866**	**21.314**	**4.938**	**Malignancy**	**NSCLC-AC**	**LC**	**Yes**	**2**
**154**	**LC97**	**LCG**	**0.640**	**30.316**	**5.061**	**Malignancy**	**NSCLC-SCC**	**LC**	**Yes**	**2**
**155**	**LC101**	**LCG**	**0.886**	**124.140**	**7.134**	**Malignancy**	**NSCLC-AC**	**LC**	**Yes**	**2**
**156**	**LC111**	**LCG**	**1.038**	**135.680**	**7.407**	**Malignancy**	**NSCLC-SCC**	**LC**	**Yes**	**2**
**157**	**LC89**	**LCG**	**0.593**	**174.337**	**7.139**	**Malignancy**	**NSCLC-SCC**	**LC**	**Yes**	**2**
**158**	**LC105**	**LCG**	**0.451**	**100.847**	**6.183**	**Malignancy**	**PC**	**LC**	**Yes**	**2**
**159**	**LC101**	**LCG**	**17.513**	**1.410**	**4.689**	**Malignancy**	**NSCLC-AC**	**LC**	**Yes**	**3**
**160**	**LC111**	**LCG**	**69.698**	**0.300**	**4.203**	**Malignancy**	**NSCLC-SCC**	**LC**	**Yes**	**3**
**161**	**LC89**	**LCG**	**1.385**	**0.305**	**0.182**	**Malignancy**	**NSCLC-SCC**	**LC**	**Yes**	**3**
**162**	**LC105**	**LCG**	**42.294**	**1.037**	**5.220**	**Malignancy**	**PC**	**LC**	**Yes**	**3**
**163**	**LC92**	**LCG**	**60.808**	**5.927**	**7.744**	**Malignancy**	**NSCLC-AC**	**LC**	**Yes**	**3**
**164**	**LC95**	**LCG**	**1.626**	**0.257**	**0.138**	**Malignancy**	**NSCLC-SCC**	**LC**	**Yes**	**3**
**165**	**LC53**	**LCG**	**55.022**	**1.048**	**5.504**	**Malignancy**	**NSCLC-AC**	**LC**	**Yes**	**3**

**166**	**LC84**	**LCG**	**339.14 3**	**0.219**	**5.451**	**Malignancy**	**NSCLC-AC**	**LC**	**Yes**	**3**
**167**	**LC97**	**LCG**	**5.649**	**0.227**	**1.270**	**Malignancy**	**NSCLC-SCC**	**LC**	**Yes**	**3**
